# Perioperative Quality Initiative consensus statement recommendations on the definition, development, implementation and outcomes of pre‐operative surgery schools

**DOI:** 10.1111/anae.16648

**Published:** 2025-06-21

**Authors:** Imogen Fecher‐Jones, Ben Ainsworth, Tong J. Gan, S. Ramani Moonesinghe, Andrew D. Shaw, Michael P. W. Grocott, Denny Z. H. Levett, the Perioperative Quality Initiative Surgery School Consensus Group, Anna M Anderson, Leah Avery, Gabriele Baldini, Angie Balfour, Rachael Barlow, Esther Carr, Daniel Conway, Robert J Copeland, Anh Dang, Andrew Davies, Mark R Edwards, Rochelle Furtado, Chelsia Gillis, Hilmy Ismail, Sandy Jack, Carol Keen, Ruth McDonald, Scarlett McNally, Zoe Merchant, Claire Moore, John Moore, Judith Partridge, Jashvant Poeran, Brocha Z Stern, Janius Tsang

**Affiliations:** ^1^ Department of Perioperative Care University Hospital Southampton NHS Foundation Trust Southampton UK; ^2^ School of Psychology, Faculty of Environmental and Life Sciences University of Southampton Southampton UK; ^3^ Division of Anesthesiology, Critical Care and Pain Medicine University of Texas Austin Texas USA; ^4^ Division of Anesthesiology MD Anderson Cancer Center Houston TX USA; ^5^ NIHR University College London Hospitals Biomedical Research Centre, University College London Hospitals London UK; ^6^ Department of Targeted Intervention University College London London UK; ^7^ Department of Intensive Care and Resuscitation Cleveland Clinic Cleveland OH USA; ^8^ NIHR Southampton Biomedical Research Centre, University Hospital Southampton NHS Foundation Trust/University of Southampton Southampton UK; ^9^ NIHR Southampton Biomedical Research Centre, University Hospital Southampton NHS Foundation Trust/University of Southampton Southampton UK

**Keywords:** Delphi consensus, peri‐operative medicine, pre‐operative education, surgery school, prehabilitation

## Abstract

**Introduction:**

Pre‐operative group sessions incorporating patient education and behaviour change interventions, known as ‘surgery schools’, are becoming increasingly common before major elective surgery across the world. However, there is a lack of conclusive evidence regarding the effectiveness of surgery schools, and the development and delivery of these complex interventions lacks standardisation.

**Methods:**

In collaboration with the Perioperative Quality Initiative, we aimed to develop evidence‐ and expertise‐based consensus statements and recommendations regarding the definition, design, content, and outcomes of surgery schools. Thirty‐two international multidisciplinary experts in surgery school and pre‐operative preparation attended a series of virtual meetings based on a modified Delphi methodology. A systematic review and additional targeted literature searches were used to propose statements for the definition, design, content and outcomes of surgery schools. Statements and recommendations were discussed iteratively and refined in multiple rounds, until agreement was reached.

**Results:**

Consensus was reached on a definition of surgery school, as well as three statements and 18 recommendations in relation to: scope; outcomes; intervention development; delivery; inclusivity; and educational content of surgery schools. Seventeen areas were highlighted as priorities for future research.

**Discussion:**

These consensus statements and recommendations are intended to help clinicians and service managers who plan to develop and implement surgery schools. They may improve the quality of those programmes and help to standardise their content. We also hope that this work will influence government strategy and policy in relation to the design, delivery and funding of peri‐operative optimisation pathways.

## Plain Language Summary

Before big, planned operations, many hospitals now run special group classes called ‘surgery schools.’ These help patients learn what to expect and how to get ready for their operation. But doctors still don't know for sure how well these classes work, and there is no clear guide as to how they should be set up. To help fix this, a group of experts from around the world worked together. They wanted to agree on what surgery schools should look like and what they should teach. They met online in meetings and looked at lots of studies and research. They talked through ideas in several rounds until they all agreed on the best advice. The team agreed on a definition of what a surgery school is and produced 21 pieces of advice about how to plan and run these schools. These included what should be taught, how to make the classes easy for everyone to join and how to deliver them well. They also found 17 topics that need more research. These new ideas can help doctors and hospital staff create better surgery schools. This might make the classes more useful and more similar across different hospitals. The team also hopes their work will help guide health leaders when they make rules and decisions about these programmes.

## Introduction

Group pre‐operative patient education, known as ‘surgery school’, has been discussed in the international peri‐operative literature for over 50 years [1]. This has traditionally been delivered by clinicians to patients and their families and/or carers before major elective surgery, with the aim of preparing them for surgery and optimising their recovery [2]. Randomised controlled trials suggest that patients who attend surgery school (compared with those who do not) have shorter durations of hospital stay; lower levels of pre‐operative anxiety and self‐reported postoperative pain; and an improved postoperative quality of life [3, 4, 5]. However, there is currently no precise definition of a ‘surgery school’ despite them being implemented in multiple surgical specialities including orthopaedic; cardiac; gastrointestinal; and gynaecological surgery. Interventions vary considerably [2] and lack of consistency in reporting content hinders replicability. The diversity of reported outcomes also makes drawing conclusions on the effectiveness of surgery schools challenging [6].

The introduction of peri‐operative pathways and interventions such as enhanced recovery, peri‐operative medicine and prehabilitation has transformed surgical preparation and the recovery process. The drive to empower patients to take control of their own preparation for surgery and make lifestyle behaviour changes proactively is reflected in the exponential rise of published work supporting surgery schools in the last decade [6]. This, together with the known benefits of group interaction and psychosocial support in relation to behaviour change [7, 8], makes a compelling case for all patients to be offered pre‐operative group education before major elective surgery. This sentiment is endorsed by professional societies including the UK Centre for Perioperative Care [9].

Whilst work has been undertaken to establish consensus on group education before total knee replacement surgery [10], there remains an urgent need for international consensus on the components and content of surgery schools applicable to all specialities. This is likely to lead to a more standardised approach to surgery schools as an education and behaviour change intervention, based on the best available evidence and expert opinion.

## Methods

The AGREE 2 Instrument [11] and supporting AGREE Reporting Checklist [12] were used to guide the methodology and reporting of this consensus study. Both are used widely and aim to improve rigour and transparency in the reporting of practice guidelines.

The Perioperative Quality Initiative (POQI) is an international multidisciplinary non‐profit organisation that organises consensus conferences on clinical topics related to peri‐operative medicine and surgery [13]. Each POQI conference brings together a collaborative and diverse group of experts from multiple healthcare disciplines to develop consensus‐based recommendations in peri‐operative medicine or surgery using an approach first described in detail by Kellum et al. [14], and described subsequently as a ‘modified’ Delphi approach in published POQI papers [15]. This approach involves alternating whole group plenary sessions and small workgroups with critical appraisal of statements/recommendations in plenary sessions and iterative refinement during workgroups.

The POQI process was identified as a suitable approach for this work as it is an established method for generating evidence‐based guidance using robust methodology [16]. The strength of the POQI process is that it acts as an interface between evidence and clinical expertise as well as identifying clear recommendations for future research. Because this consensus process was conducted via remote online methods, small modifications were made to the methods of Kellum et al. [14], as described below. Three members of the POQI Board were present at all the workshops to moderate and ensure that the POQI‐modified Delphi approach was adhered to.

Thirty‐six experts (including the three members of the POQI Board) were identified by reviewing relevant literature and through pre‐existing professional networks and academic societies. They were then invited via email to attend four surgery school consensus meetings. Attendance was required at all four meetings (12 hours in total), which took place over a 2‐month period using Microsoft Teams (Microsoft Corp, Redmond, WA, USA).

The preparatory work and meetings followed the broad stages of a Delphi process (Table 1). A systematic review of pre‐operative surgery schools, as well as additional targeted literature reviews on each topic, were conducted by IF‐J [6] to identify relevant evidence for best practice. Initially, 30 candidate statements and recommendations for discussion were proposed by IF‐J; a ‘statement’ was defined as a position supported by the literature, and a ‘recommendation’ was defined as a suggested course of action based on the literature and expert opinion. These were accompanied by evidence summaries together with a suggested level of certainty of the supporting evidence using the GRADE approach (a scale ranging from very low to high, based on the authors' confidence in the evidence) [17] and an overall strength of recommendation (see online Supporting Information Appendix S2).

**Table 1:** Meeting schedule aligned to consensus stages.

Consensus stages	Activity
Defining the problem	Systematic review, targeted searches
Develop candidate statements and recommendations	Live working document including evidence summaries
Establish expert group	Preliminary meeting: Introduction of proposed draft statements and recommendations. Explanation of GRADE and POQI rules
Round 1 (workshop 1)	Candidate statements and recommendations presented in a plenary session, followed by discussion and revision until agreement reached in two smaller groups based on topic and expertise. Group agreement on each statement/recommendation was ascertained through raising a virtual ‘thumbs up’ which was monitored by group chairs. Final version is agreed upon
Post‐meet analysis	Recording of meeting and chat comments reviewed to further analyse responses, identifying trends and points of disagreement for next meeting
Round 2 (workshop 2)	Revised statements and recommendations presented in a plenary session, followed by discussion and voting as above in two smaller groups based on topic and expertise
Post‐meet analysis	Recording of meeting and chat comments reviewed to further analyse responses, identifying trends and points of disagreement for next meeting
Round 3 (final meeting)	Final voting on wording, grading and strength of recommendation of statements and recommendations
Final analysis and reporting	Final statements and recommendations circulated via email to confirm agreement. Publication manuscript reviewed and contributed to by all members

A leadership team comprising IF‐J, MG and DL met to discuss the proposed statements and recommendations for discussion and compile them within a live working document. This document was added to continually and edited throughout each of the expert meetings and during the analysis which took place in between meetings.

The degree of group agreement on each statement/recommendation during the workshops was ascertained through Roman voting (one thumb vote) using the Microsoft Teams reaction functions. Voting was moderated by group chairs and later verified by IF‐J following review of each meeting recording and transcription. During refinement of statements/recommendations, 85% agreement was required before moving on to the next statement or recommendation. If agreement was not reached, the statement/recommendation was either developed further until agreement was achieved or was removed. At the end of the final session, approval or dissent was sought for the final set of recommendations. To verify agreement, after the final meeting, the final versions of the definition, statements and recommendations were circulated via email, and written confirmation of agreement was received from all participants. Full detail on conference structure can be found in online Supporting Information Appendix S3.

Until recently, POQI methodology has not tended to include patient and public involvement (PPI), as recommendations have traditionally been based purely on the published evidence and clinical expertise. However, as surgery schools are an intervention for patients, the group agreed that it was important to seek patient input. Before the final session, IF‐J took the draft final statements and recommendations to a PPI group consisting of five people of mixed ages, sex and ethnicity, who had undergone major surgery themselves. Each statement was presented and discussed. Feedback from the PPI group was presented to the expert group during the final meeting and included in the final statements.

## Results

Of the 36 invited experts, 32 agreed to participate as POQI Surgery School Consensus Group members. Twenty‐three of these participants had published work relating to surgery schools; the remaining nine had published in the fields of prehabilitation or pre‐operative education, and most had practical experience of delivering pre‐operative education in clinical practice. The professional backgrounds of participants included: anaesthetists; surgeons; nurses; physiotherapists; occupational therapists; dietitians; clinical scientists; psychologists; a geriatrician; and an epidemiologist. Participants' professional practice was based in the UK; Canada; USA; Italy; and Australia (online Supporting Information Appendix S4 details expert demographics and characteristics).

**Figure 1:**
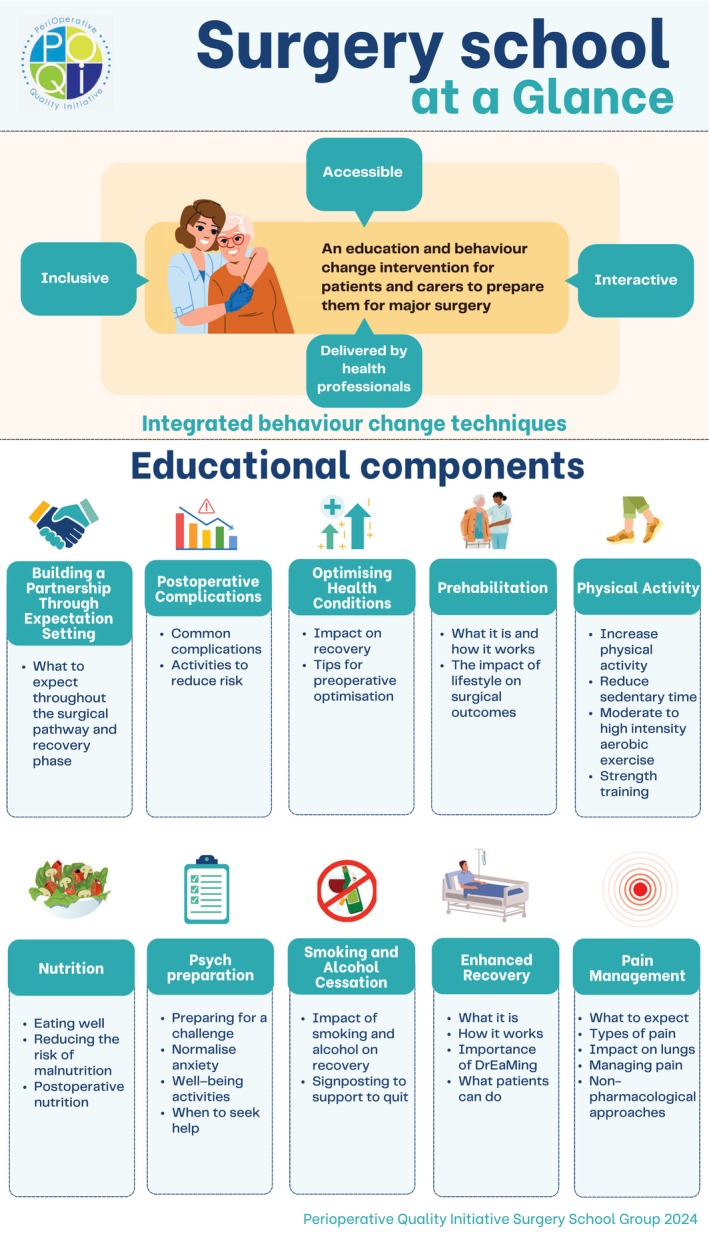
Surgery school at a glance. Image reproduced with permission of the Perioperative Quality Initiative. DrEaMing: drinking, eating and moblising.

Throughout the 12 hours of meetings, the original 30 proposed statements and recommendations were refined to a definition, three statements and 18 recommendations (Fig. 1). These all achieved at least 85% agreement through the voting process outlined above. The remaining eight recommendations were unable to achieve this level of agreement and were therefore not included. When initial GRADE recommendations were challenged, this typically resulted in a statement or recommendation being downgraded to ensure the GRADE and strength of recommendation reflected the level of evidence available. At the end of the process, unanimous approval was achieved for the final version of the definition, statements and recommendations.

### Definition of surgery school

A definition of ‘surgery school’ was developed, based on the elements described most commonly within the literature: ‘*Surgery school is an education and behaviour change intervention, delivered by healthcare professionals to groups of patients and their family, friends and carers, which aims to prepare them for major surgery*’.

The group was unanimous about the aims and the importance of surgery school being an interactive and engaging experience for the patient and their support network, which was ultimately defined as ‘family, friends and carers’. Inviting support networks to attend alongside the patient serves to improve the likelihood of success when carrying out preparation activities and, in particular, behaviour change [18], such as increasing physical activity or making dietary changes. The group agreed that surgery schools should be delivered in partnership with patients and their caregivers. The aim is to create the conditions that help educate, enable and empower patients to take control of their preparatory journey and postoperative recovery, which is known to improve adherence and engagement with treatment pathways [19]. The notion of surgery schools not being an isolated event, but a gateway to the peri‐operative path ahead, was also endorsed strongly by the PPI group, which perceived surgery school as a pre‐operative induction programme with a central theme of empowerment.

### Outcomes of surgery schools

It was agreed by the expert group that consensus statements should be made in relation to the outcomes of surgery schools. For this purpose, ‘clinical outcomes’ were defined as those relevant to surgery (including duration of hospital stay; morbidity; mortality; and readmissions [20]) and ‘patient‐centred outcomes’ were defined as outcomes that are meaningful, valuable and helpful for patients who have experienced surgery school [21] (including knowledge; anxiety; preparedness; pain; physical function; quality of life; and satisfaction).

There was agreement that there was a stronger evidence base (‘high level of certainty’ according to GRADE) for ‘satisfaction’ and ‘experience’ than the other patient‐centred outcomes. In quantitative studies, increase in satisfaction applies specifically to patients' experience of attending surgery schools and the outcomes of surgery [3, 5, 22, 23, 24]. The other outcome statements were agreed to be of ‘moderate level of certainty’ due to the heterogeneity of the studies that provide supporting evidence for the statements; further high‐quality studies are needed in this area.

- Surgery schools may improve clinical outcomes from major surgery (moderate level of certainty, statement)
- Surgery school may improve patient‐centred outcomes including physical function, comfort, anxiety, quality of life and preparedness (moderate level of certainty, statement).
- Surgery schools are well accepted by patients who attend, increase patient satisfaction and improve patient experience (high level of certainty, statement).

### Who should attend surgery schools?

Based on the clinical and patient‐centred outcomes evidence, the group agreed with the Centre for Perioperative Care recommendation that all patients undergoing major elective surgery should be offered the opportunity to attend surgery school [9]. However, all of the members of the expert group were from high‐income countries, and it was acknowledged that surgery schools may not be feasible on a global level, although there is some evidence of provision in some middle and low‐income countries [25, 26].

For the purposes of this recommendation, ‘major surgery’ was defined as any elective surgery normally requiring more than one night's stay in hospital, as most surgery school studies fit this description. However, given the international context, it was recognised that some major surgeries (e.g. total knee replacement) are transitioning to same‐day discharge in some centres but would still fit within this classification in terms of the relevance of surgery school. All surgical specialties are included as most, if not all, are represented in the surgery school literature. It was acknowledged that some areas such as orthopaedics, transplant and gender affirming surgery may be more appropriate as single specialty schools [10, 26, 27]. However, there is also a growing case for surgery schools being mixed specialty [28, 29, 30] as this enables more efficient planning and use of healthcare resources.

It was highlighted by the PPI group that surgery schools may increase anxiety for a small subset of patients, which is also supported in the literature [31]. However, no study has identified any major risk of harm in attending surgery school, and it was agreed that the benefits of attending are likely to outweigh any risk.

- All patients having major elective surgery should be offered and advised to attend ‘surgery school’ (moderate level of certainty, strong recommendation).

### Components of surgery school

In line with the definition from the Medical Research Council [32], surgery schools should be considered a complex intervention as they are a multicomponent healthcare intervention that targets a range of behaviours. The intended audience and context for delivery also vary widely, with national and international variance meaning that surgery schools may require local modifications to suit the local population and context.

With the growing evidence base and drive for prehabilitation before surgery [33], the role of surgery schools in promoting lifestyle behaviour change has become a key focus. As with any behaviour change intervention, due consideration must be given to development, implementation and evaluation.

The use of theoretical models and frameworks is recommended to support identification of target behaviour determinants, outcomes and key components of surgery schools intended to improve their effectiveness [34]. One tool that can be helpful in theory building and developing an understanding of how an intervention will produce an intended outcome, is a ‘logic model’. Applying the process of building a logic model for surgery school will help ensure the objectives are aligned with the most appropriate mechanisms of action to achieve desired behavioural changes and ultimately patient outcomes. A specific logic model or intervention map that could be applied to a surgery school was beyond the scope of this consensus; however, there are specialty‐specific examples available in the literature [35, 36].

During the design of surgery schools, consideration should be given to how desired behaviour changes may be achieved. This will involve selecting specific evidence‐informed behaviour change techniques [37] embedded within the curriculum's supporting materials and including techniques such as goal setting and guidance on habit formation [38]. The most cited behaviour change techniques used within surgery schools and similar interventions targeting lifestyle behaviour change are shown in online Supporting Information Appendix S5. There is little evidence to suggest that these techniques are associated with effectiveness; however, they are arguably the most frequently reported and easiest to implement within this type of intervention, and consequently there is a risk of bias for their use.

Following the implementation of a new surgery school, evaluation should be undertaken to ensure it is producing the desired effects as described in the logic model. The most measured outcomes of surgery school are duration of hospital stay, complications and patient experience or satisfaction [6]. However, consideration should also be given to other meaningful outcomes and process measures for organisations and patients, including cost‐effectiveness; changes in behaviours; patient activation; or self‐efficacy [39].

- Surgery schools are a complex intervention and should be designed, implemented and evaluated using established theoretical models and should incorporate behaviour change techniques (high level of certainty, strong recommendation).

Together with targeting behaviour change, enabling an increase in self‐efficacy (a person's confidence that they will achieve their goal despite barriers, e.g. adopting health‐promoting behaviour) within surgery school is recommended. Self‐efficacy is considered one of the most important prerequisites for a wide range of health behaviour changes [40]. Patients with higher levels of self‐efficacy are much more likely to make behavioural changes that will potentially improve their surgical outcome [41]. Many of the behaviour change techniques cited most commonly, such as goal setting and verbal persuasion, can also be effective in increasing self‐efficacy [42] and therefore should be considered when identifying which behaviour change techniques are the most appropriate to use within surgery school for the target population. Early work on increasing self‐efficacy [43] highlights the importance of observing relatable others succeeding in activities. Educational materials should therefore include images and stories of other patients in similar circumstances who have successfully adopted health‐promoting behaviours.

While components aimed at increasing self‐efficacy can be embedded in surgery schools, it was acknowledged that improving self‐efficacy is not normally achieved fully through a single interaction and requires reinforcement [44]. Most patients are likely to require further support to increase their activation levels. The importance of signposting to appropriate agencies who may be able to provide this support is therefore essential.

- Surgery schools should introduce strategies to improve patient self‐efficacy to adopt health promoting behaviours (high level of certainty, strong recommendation).

### Design and delivery of surgery school

As multicomponent behaviour change interventions cover a wide range of topics, surgery schools should be developed by relevant members of a multidisciplinary team. Most commonly in practice this includes anaesthetists; nurses; physiotherapists; occupational therapists; and dietitians [2, 10]. Involving a psychologist or behaviour change expert in the design phase is also recommended given the key behaviour change elements. Patients should also be involved in the design of surgery schools, particularly those with experience of surgery. These findings are in line with a previous consensus study by Anderson et al., which involved a 60‐member expert panel and suggested pre‐operative total knee replacement education should be informed by patients and multidisciplinary professionals [10].

Patients report that having a variety of educators created feelings of trust and respect [45], that they enjoyed these interactions and it made the school more engaging [29]. Having information delivered by a credible source is a known and commonly used behaviour change technique [37]; patients have reported that their adherence to prehabilitation was motivated mainly by the fact it was recommended by their doctor [46]. Surgery schools are a unique opportunity for collaborative working that has direct benefits for patients who are receiving the right information from the right people at the right time.

The financial implications of having multiple professionals deliver surgery school together is a concern in many healthcare systems. Surgery schools should be delivered only by those with ‘expertise in the content matter’, which lessens the focus on specific professions. The potential for a defined competency framework for those delivering surgery school was considered but not thought to be necessary providing the expertise element of this recommendation was adhered to.

- Surgery schools should be developed by a multidisciplinary team and delivered by individuals with content matter expertise (high level of certainty, strong recommendation).

The 23 group members with firsthand experience of delivering surgery schools believed that it is critical for them to be: understandable; interactive; and live. This is in line with the findings of the consensus study by Anderson et al., in which 89% of respondents rated delivery of pre‐operative total knee replacement education in group face‐to‐face sessions as ‘important’ or ‘very important’ [10]. Our PPI group also highlighted interactivity as an essential component of surgery schools. Among our experts there was unanimous agreement that live sessions, whether in‐person or virtual, promoted patient engagement and interactivity, but that care should be taken during intervention development to ensure that education delivered was easily digestible and understandable to promote self‐efficacy. The ability to bring family/friends/carers along was also considered important in promoting engagement.

The experts who delivered surgery school reported the benefit for patients of being in a group with other patients, a concept which is supported by qualitative literature. Patients report feeling a sense of social connectedness [46], as well as comfort and enjoyment from meeting others in the same situation [29, 31, 47]. Within the wider literature it is reported that group delivery can support the behaviour change elements and is an effective way of delivering behaviour change interventions [7, 8].

Provision of supporting information for patients to digest in their own time was also considered essential by the expert and PPI groups. Supporting information was defined as any information that covered the key learning points of surgery school and should include written information in the form of leaflets but may also include other media such as online material and DVDs.

- Surgery schools should be easily understandable, interactive, delivered live (virtually or in‐person) and include supporting material (low level of certainty, strong recommendation).

Whether in‐person or virtual, surgery schools will not be acceptable to all patients and by their definition will create some barriers to attendance [29, 48]. Measures should be taken to ensure that all patients receive appropriate comparable pre‐operative education and preparation in an acceptable format to promote health equality. Careful consideration should be given in the design, development and delivery of all surgery school educational materials to ensure that they meet the needs of patients with any disability, impairment or sensory loss, and other considerations (e.g. minority language) [49]. Patients with disabilities should be made aware of what reasonable adjustments they can request to minimise any barriers to engagement and how to request them, to help ensure they have equality of access to surgery schools and associated materials. Universal health literacy precautions should be integrated to promote understanding to all [50].

If surgery school is delivered digitally then support should be available to patients such as clear instructions and real‐time support on how to access online applications, or loan of a digital device on which to take part. Patients who are unable to attend surgery school should be offered an alternative such as a one‐to‐one consultation or access to an asynchronous recording. Alternatives to any written material such as videos should be considered as they are likely to be more accessible to patients with lower literacy levels [51]. The challenge of being able to provide something for everyone within short waiting timeframes and limited financial resources was acknowledged, highlighting the need for national bodies to produce surgery school supporting resources such as ‘Fitter Better Sooner’ [52].

Lastly, it was acknowledged that highlighting unhealthy behaviours and the associated impact on risk may result in patients feeling stigmatised, particularly if they have low self‐efficacy to enact any behaviour change. Actions to mitigate this included patient involvement in development of materials, using a ‘solution focused’ approach to communicating advice [53], with the aim of generating hope and confidence rather than despair. Materials should avoid using visual images featuring stereotypes and be delivered with professionalism and empathy. Clear signposting for further support should also be included.

- Providers should promote inclusivity to reduce known health inequalities throughout the design and implementation of surgery schools (high level of certainty, strong recommendation).

### Educational content of surgery school

These recommendations cover what educational content was agreed to be most useful within surgery school. A summary infographic is presented in Fig. 1 and a full breakdown of the suggested content for each topic is included in online Supporting Information Appendix S6.

Setting patients' expectations was considered an essential content element of surgery schools. Patients who understand what to expect during their surgical journey have lower levels of anxiety [54] and are more likely to adhere to recovery programmes [55]. When expectations are realised, patients have lower levels of postoperative anxiety [5, 56] and higher levels of satisfaction [23, 24] and there is an association with fulfilment of expectation and patient‐reported outcomes [57].

Fostering the concept of a partnership between health professionals and patients was agreed to be essential. A feeling of partnership during preparation for surgery and recovery has been described by patients as empowering [58] and, according to the theoretical framework of Pellino et al., is likely to lead to an increase in self‐efficacy and patients actively making behaviour modifications [59]. Within the literature, patient empowerment is used synonymously with patient activation, and there is an acceptance that these more ‘activated’ individuals are more likely to make the most of the ‘teachable moment’ as there is already a ‘readiness’ for change [60]. Patients require knowledge to be equal partners in their preparation and recovery [55]. A partnership is generated through the sharing of knowledge about what patients can expect to experience, whilst concurrently highlighting the patients' role and supporting them to take control of their preparation and prehabilitation for surgery.

- Surgery schools should help set expectations and develop a partnership with patients in preparation for and recovery from surgery (moderate level of certainty, strong recommendation).

Long‐term health conditions affect one in four surgical patients and increase the risk of postoperative death significantly [61]. Surgery school therefore presents an opportunity to highlight the importance of optimising long‐term health conditions before surgery. Due to the lack of published evidence on this topic, the agreed evidence grade for this recommendation is low. However, there is a growing understanding in practice regarding chronic disease conditions and the impact on surgical outcomes [62], which supports this being a strong recommendation. Conditions of particular relevance include diabetes mellitus [63]; hypertension [64]; cardiac disease [65]; respiratory disease [66]; anaemia [67]; and chronic pain [68], but this list is by no means exhaustive.

- Surgery school should support patients to understand the importance of optimising long‐term health conditions prior to surgery and signpost to appropriate resources (low level of certainty, strong recommendation).

In a longitudinal study of 697 patients having elective surgery [69], patients reported that postoperative complications were the topic above all others that they wanted more information about. It is known that the impact of surgical complications is often underestimated by patients [70], can be life‐changing [71] and, importantly, in some cases can be mitigated by patients taking precautionary actions [72, 73]. Understanding the risks of the most common surgical complications was also agreed to be important knowledge by Anderson et al. in their consensus on the content of pre‐operative education [10]. Receiving this knowledge is also likely to help motivate patients towards making behaviour changes [74].

Which specific peri‐operative complications to include will be dictated by whether the surgery school is mixed or single specialty. However, as a minimum, the most common surgical complications (pulmonary, infection, bleeding, thrombosis, renal injury and delirium) should be mentioned, with particular attention drawn to those for which there are modifiable risk factors such as pulmonary infection; venous embolism; bleeding; and infection.

Due to the strong evidence for the impact of patient education on reducing the incidence of pulmonary complications [28, 72, 75], it was agreed patients should be provided with specific advice on how they can reduce this risk. These activities are likely to be surgery specific but, as a minimum, should include breathing techniques such as the Active Cycle of Breathing [76]. Explanation of inspiratory muscle training could also be considered [77]; demonstration of these techniques and, where possible, participant practice is more likely to lead to adherence to pre‐ and postoperative breathing exercises [78]. However, it was recognised that if spirometry devices are to be used, they are not suitable for group training because the device must be calibrated to the individual.

- Surgery schools should support patients to understand common postoperative complications (moderate level of certainty, strong recommendation).
- Surgery schools should inform patients about the risks of pulmonary complications and give advice on activities to reduce this risk (high level of certainty, strong recommendation).

Four recommendations relate to prehabilitation education. Prehabilitation is a process of enhancing functional capacity and physiological reserve to allow patients to withstand surgical stressors with the aim of improving postoperative outcomes and facilitating recovery [79]. It is generally accepted that prehabilitation should consist of one or more pre‐operative interventions of exercise; nutrition; psychological strategies; and respiratory training [79]. Although most of the evidence for improved surgical outcomes is based on patients having surgery for cancer, promising evidence suggests that patients undergoing a wide variety of surgeries stand to benefit. Within peri‐operative care, it is recommended that prehabilitation services incorporate the multimodal elements of exercise, nutrition and psychological wellbeing, and aim to deliver a personalised approach [80, 81]. Patients should first undergo screening to identify their level of need. However, currently, this is not feasible in many organisations due to the additional resources necessary to deliver prehabilitation services.

Within the expert group it was agreed that patients should be advised to reduce sedentary time and increase their ‘activity levels’. Specific examples of aerobic activities, with a focus on effort and duration, should also be provided. Some concerns were raised regarding the safety of advising all patients attending surgery schools to do moderately intense activities. However, it was ultimately agreed that this level of intensity is supported by World Health Organization recommendations [82] as well as campaigns such as Move More Sheffield [83], and that there was greater risk to patients in doing nothing than doing something [84].

The need to integrate behaviour change techniques, such as goal setting and self‐monitoring [78], within taught content was also discussed as essential in supporting patients to make behaviour changes, e.g. increasing physical activity.

Nutritional status before and after surgery is a modifiable and recognised element of multimodal prehabilitation, and has been shown to improve surgical outcomes [85]. Giving universal‐level recommendations, including public health recommendations for healthy eating and clinical nutrition recommendations for surgery [86], may reduce the risk of peri‐operative malnutrition [87]. This includes eating balanced meals; consuming adequate protein as well as fibre from fruit and vegetables; and reducing intake of ultra‐processed foods associated with poor nutrient density and obesity. Patients should also be taught how to self‐screen for risk of malnutrition [86], and be provided with tips on managing common pre‐ and postoperative nutritional impact symptoms (e.g. poor appetite and nausea), which are known to increase the risk of peri‐operative malnutrition [88].

Some patients attending surgery school may not be able to reduce their risk of malnutrition without targeted or specialist support [89]. Consideration should therefore be given regarding direct referral/signposting to local nutrition professionals for appropriate levels of intervention for those identified as at risk [90], as well as providing information on additional support services such as food banks, box schemes and community kitchens.

Although the quality of evidence is low, it was agreed that psychological preparation, as the third recognised element of multimodal prehabilitation [91], should be included within surgery schools. However, whilst there is some evidence to suggest psychological factors impact surgical outcomes [92] there is little available guidance regarding how best to prepare psychologically for surgery [93].

Surgery schools have been shown to reduce pre‐operative anxiety when compared with a baseline or control group [5, 25, 46, 56], and this appears, in part, to be due to improved insight into what to expect. The group agreed that in addition to setting expectations, patients should also be primed mentally to prepare for a challenge. Having surgery should be acknowledged as a major life event, but not one that is insurmountable despite the mental and physical challenges it may present. Preparing for this challenge should be framed in a supportive and positive way to avoid inducing health anxiety, foremost by supporting patients to understand that they are not alone and will be supported to get through it. This partnership element is also crucial in building psychological resilience [94].

As well as general psychological preparation, surgery school providers may wish to consider giving advice on how to recognise and manage depression and anxiety (including seeking professional intervention). Consideration could also be given to providing strategies for how patients can optimise and maintain their wellbeing by doing regular exercise [95], taking part in relaxation activities [96] and avoiding known depressants such as alcohol [97]. It was acknowledged that, as with the other prehabilitation elements, there would be a proportion of patients who would require more specialist services; therefore, signposting to where patients could receive additional psychological support should also be considered.

- Surgery schools should support patients to understand the principles of multimodal prehabilitation and the impact of health behaviours on surgical outcomes (moderate level of certainty, strong recommendation).
- Surgery schools should encourage and support patients to plan and undertake physical activity and exercise (moderate level of certainty, strong recommendation).
- Surgery schools should emphasise the importance of good nutrition before and after surgery and signpost appropriate resources (high level of certainty, strong recommendation).
- Surgery schools should support patients to prepare psychologically for surgery (low level of certainty, strong recommendation).

Alcohol use disorder (increasing risk, higher risk and dependent pattern drinking [98]) has a negative impact on surgical outcomes [99]. There is some suggestion that a ‘brief’ educational intervention alone may be enough to support patients towards cessation [73], leading to many surgery schools including this element within their programme [2]. Similar expert consensus within the orthopaedic surgery literature also recommends inclusion of this topic [10]. Surgery schools may provide an opportunity for patients to self‐screen their risk level using a validated tool [100], as some may be unaware that their drinking habits are unsafe. Those who identify themselves as ‘at risk’ should be signposted towards additional support and/or specialist alcohol services where available. However, self‐screening in a group setting may lead to underestimation of alcohol intake and, due to the sensitive nature of the topic, patients should not be asked to share their results. It was agreed that due to a lack of evidence regarding a ‘safe’ amount of alcohol before surgery, all patients should be advised not to drink before surgery. The optimum duration of abstinence is not absolutely clear, but is likely to be between 2 and 4 weeks [101].

Due to the risk of withdrawal symptoms for those who typically drink over 15 units of alcohol per day, patients who self‐screen as to as ‘high risk’ should be advised as such and encouraged to self‐refer or at least be signposted to specialist alcohol services regarding community‐based assisted withdrawal [102]. It should be highlighted that alcohol cessation for these patients should be gradual.

- Surgery schools should inform patients about the risks of excessive alcohol consumption and support patients to limit alcohol intake before surgery (moderate level of certainty, strong recommendation).

Pre‐operative tobacco usage increases the risk of postoperative complications [103], which has led to national consensus on providing smoking cessation guidance for patients before surgery [104]. As with alcohol, smoking cessation advice is already included within many surgery schools. Patients should be advised on the reasons they should stop smoking before surgery by having the risks and benefits of doing so presented to them. It should also be made clear that smoking includes inhalation of any substance that contains nicotine, including cigars. Discussion around the potential risks of vaping [105] should also be included. Patients who smoke should be signposted to smoking cessation services to increase their likelihood of quitting successfully [106].

- Surgery schools should inform patients about the benefits of stopping smoking before surgery and support patients to stop smoking (high level of certainty, strong recommendation).

The likelihood of pain following surgery is one of the common causes of pre‐operative anxiety for many patients [107] and is one of the topics on which they most want information [108]. Given that anxiety is associated with increased perceived pain following surgery [109] and the potential impact of pain on lung function [110], surgery schools should include information to support patients in understanding how their pain will be managed around the time of surgery. Managing patient expectations regarding pain and setting realistic goals for pain treatment are key to effective pain management [111].

- Surgery schools should support patients to understand how their pain will be managed around the time of surgery (moderate level of certainty, strong recommendation).

Given the evidence for the benefits of enhanced recovery after surgery (ERAS) across most surgical specialties [112], and the particular benefits regarding early drinking, eating and mobilising (DrEaMing) [113], it was agreed that this was an essential component of surgery schools. Due to the strength of the evidence, this is presented as a specific recommendation. Studies have identified that patients who had a better understanding of ERAS and its importance were more likely to follow the expected programmes [55]. There is a strong association between ERAS programme compliance and patient outcomes [114], and therefore patients should be informed and understand their role in the certain patient‐led elements of ERAS such as drinking, eating and mobilising.

- Surgery schools should inform patients about the principles of ERAS and their role in early drinking, eating and mobilisation (DrEaMing) (high level of certainty, strong recommendation).

Over the course of the meetings, several unanswered research questions were identified relating to this topic (see Box 1).

Box 1 **Recommendations for study questions to be addressed in future research**

- Can surgery schools improve clinical outcomes?
- Are surgery schools cost‐effective?
- Do surgery schools result in long‐term behaviour change?
- What is the role of surgery school in minor surgery?
- Are there any risks to attending surgery school?
- Do mixed specialty schools impact on outcomes?
- Does surgery school improve patient satisfaction with the care they receive?
- What are the most effective behaviour change techniques to use within surgery school?
- Does the mode of delivery influence the outcome of surgery school?
- What skills and competencies are necessary to effectively deliver surgery school?
- What should be offered to patients if they are unable to access surgery school?
- What is the role of generative artificial intelligence in asynchronous surgery schools to promote behaviour change?
- How can surgery schools help to address health inequalities?
- What reasonable adjustments can be made to surgery schools to help meet the needs of patients with disabilities?
- Are surgery schools a form of ‘universal’ prehabilitation?
- How long before surgery should alcohol be abstained?
- What generic post discharge advice should be given to all patients to support rehabilitation?

## Discussion

This study has produced a definition of surgery school, three statements and 18 recommendations on their expected outcomes, design, content and delivery, generated using an established and moderated modified Delphi approach. This is the first surgery school consensus of its kind to date and benefits from the contributions of a large panel of international experts as well as patient and public contributors. The rigour and transparency of the POQI methods and the supporting evidence drawn from the literature offer strength to the outputs.

The three surgery school outcome statements were agreed by the group following the presentation of the results of a systematic review involving 27 studies [6]. The review identified that patients who had attended surgery school had a 0.70 (95%CI 0.27–1.13) day reduction in mean duration of hospital stay (seven orthopaedic studies) and a reduced odds ratio for postoperative complications following mixed specialty surgery (0.56 (95% CI 0.36–0.85), nine studies). However, only two studies within these meta‐analyses were randomised controlled trials [5, 115]. No studies to date have shown a significant difference in readmission rate for those who attend surgery schools (compared with those who do not) [3, 56, 116].

There are numerous patient‐reported outcomes which are improved among patients who attend surgery school, when compared with a baseline or control group. These include: patient knowledge [25, 26, 117]; patient activation [118]; pre‐operative anxiety [5, 21, 25, 27, 57, 119]; preparedness [46, 54, 56]; pre‐ and postoperative pain [3, 5, 22]; postoperative physical function [3, 120]; and quality of life [115, 121]. However, most of these studies were observational, and there was a significant degree of heterogeneity within the interventions and study designs.

Several studies have measured patient satisfaction following surgery school [3, 5, 21, 22, 23], and all report high patient acceptability and positive experience; patients describe their experience as empowering, reassuring and motivating. There is potential for participation bias within these studies, as is well established in other settings [122]. This is made more likely with most studies being conducted in English‐speaking high‐income countries. In addition, acceptability data are often only collected on attendees, and the preferences of those who attend may be systematically different from those who choose not to.

The variety of measured outcomes continues to challenge overall evaluation of surgery schools. Although there is a need for standardising outcome measurement, this was beyond the scope of the POQI and is suggested as an area for future research. It was noted, however, that some of the outcomes are likely to be the mechanisms for others; for example, an increase in knowledge is likely to influence anxiety levels, preparedness and patient activation. Increased patient activation is likely to lead to behaviour change and potentially a reduction in complications. These relationships make the case for a more targeted approach to understanding mechanisms behind the observed effectiveness of surgery schools within a larger programme of care.

Up until 2020, most surgery schools described in the literature were delivered in‐person to groups of patients. During the COVID‐19 pandemic, the virtual mode of surgery school delivery became commonplace, and has remained the default mode of delivery in many settings. Studies have found that virtual live schools are more practical, well accepted by patients, better attended and no less effective than in‐person interventions [30, 48, 123]. However, there is a known risk of digital exclusion for some patient and carer groups [48]. Only one study could be found evaluating an asynchronous (non‐live) surgery school which patients can do at their own pace, with some evidence of improved clinical outcomes when compared with no surgery school [124]. However, there are a number of asynchronous offerings available from other UK health providers [125]. Further research is needed to evaluate the effectiveness of this type of delivery, but in the absence of a live surgery school, it was agreed they were a viable alternative, particularly for those unable to attend a live session. However, this mode would likely require patients with lower health literacy or higher risks to follow up with a health professional, as pre‐operative information provided without the support of a health professional may result in negative outcomes [126].

Surgery school is recognised in the literature as an opportunity to introduce patients to the relatively new concept of prehabilitation and provide the skills and knowledge to enact new behaviours [127]. Several established surgery schools have integrated elements of multimodal prehabilitation successfully into their curricula, with some evidence of effect [29, 30, 128].

The importance of increasing physical fitness is the third most taught topic within UK surgery schools (after the ‘surgical journey’ and ‘enhanced recovery’) [2], and has been identified by patients to be one of the most useful [29]. However, there was debate within the expert group around physical activity vs. exercise: whether increasing physical activity (any bodily movement produced by skeletal muscles that requires energy expenditure [129]) was enough, or whether formal exercise (an activity that is planned, structured, repetitive with the aim of improving or maintaining physical fitness, performance and health [82]) was required to make a positive difference to patients. Most of the studies linking exercise to improved surgical outcomes used moderate intensity aerobic interventions, such as interval training along with resistance training [130]. As the difference between physical activity and prescribed exercise is not widely known by the general population, consensus was ultimately established that surgery school physical activity advice should be based on World Health Organization recommendations for physical activity [129].

It is acknowledged that there are likely to be barriers to developing and implementing surgery schools within organisations, particularly when financial and human resources may be restricted. The paucity of health economic evaluation data was of concern to the group. Studies to date have been inconclusive [131, 132] or highlighted the economic burden of delivering the surgery school when compared with standard care [131, 133]. Although any reduction in complications and duration of stay will lead to cost savings, it may be difficult to attribute these entirely to surgery school, as they are often delivered alongside other peri‐operative optimisation interventions such as anaemia management, prehabilitation and enhanced recovery. Evaluating cost effectiveness is therefore a recommendation for future research.

It is recognised that there are many more education topics that could have been included within this consensus; nevertheless, in the interests of pragmatism, we believe we have identified the 12 key content elements that should be included in any surgery school based on the evidence. We would encourage organisations implementing these recommendations to make relevant modifications based on the locality and the types of surgery their patients are having.

Much consideration was given to the active verbs used to describe the role of the educator in each content recommendation, whether that was to ‘support’, ‘advise’, ‘inform’ or ‘encourage’. Each verb was chosen in the context of the topic and the target endpoint, namely behaviour modifications. The meaning of these verbs should therefore be considered when implementing recommendations.

The suggestion to actively signpost patients to supplementary resources and targeted or specialist support arises in many of the recommendations. One surgery school will not fit all, and patients should be encouraged to seek out additional advice and support to help with enacting any necessary behavioural modifications. Signposting should be done using the social prescribing model [134] and using up to date links to reliable sources of information and local internal or external services.

Although the method is described as a ‘modified’ Delphi approach it does lack some of the key features of Delphi such as anonymity and formal voting. However, as evidenced by previous publications from the POQI, this consensus methodology is practical within this context, with outputs that contribute to the literature as well as practice [15, 135]. Further work is currently being undertaken to validate this methodology going forward.

Although care was taken to select a diverse group of experts, only seven of the group were recruited outside of the established professional networks of the lead authors. Despite good representation of the leading authors in this field, it remains the consensus of a limited sample of clinicians. Attempts were made to recruit health professionals from low‐ and middle‐income countries by contacting representatives from the NIHR Global Surgery Group and the Enhanced Recovery After Surgery International Society, but unfortunately no volunteers came forward. Recruitment of one or more primary care physicians via social media was also attempted but was unsuccessful. Although conducting the POQI virtually promoted ease of access for many, it did limit engagement from some international parties due to time zone incompatibility.

External review of the consensus recommendations was sought from five patient contributors. External expert peer review was not deemed necessary due to the size and diversity of the 32 expert panel contributors. However, there are many experts in the field who were not part of this process, and it is possible that their views may have differed.

No formal systematic review or meta‐analysis was included other than for surgery school outcomes, which aligned with the pragmatic nature of the methodology. A targeted approach was adopted due to the diversity of topics and is also aligned with the pragmatic nature of POQI methodology. Risk of bias was not considered on an individual study basis and decisions on GRADE were based on expert opinion on the body of evidence.

Although we have identified a lack of high‐quality effectiveness trials, the fact remains that surgery schools are becoming standard care in many parts of the world, and therefore consensus was required to establish best practice. It is hoped that the guidance generated will help healthcare staff develop their own surgery schools and lead to improved standardisation of surgery school services. If these recommendations are followed, patients will benefit from comprehensive evidence‐based advice and support and will be provided with the tools to prepare themselves more effectively for surgery and recovery. This in turn will likely improve their outcomes. This potential impact has been recognised by the NHS in England and a UK cancer charity that has already expressed an interest in utilising these findings to support patients to prepare physically and mentally for surgery. It is hoped that other international health bodies will also follow suit to facilitate standardisation of surgery school delivery.

## Supporting information


**Appendix S1.** Perioperative Quality Initiative Surgery School Consensus Group.

**Appendix S2.** Perioperative Quality Initiative grades of evidence and strength of recommendation.
**Appendix S3.** Perioperative Quality Initiative conference structure.
**Appendix S4.** Demographics and characteristics of expert group.
**Appendix S5.** Behaviour change techniques used most commonly in surgery schools.
**Appendix S6.** Suggested educational content to comply with recommendations.

Plain Language Summary
