# Supplementary material for: Perioperative Quality Initiative consensus statement recommendations on the definition, development, implementation and outcomes of pre‐operative surgery schools
Source: Anaesthesia. 2025 Jun 21;80(9):1115–33. doi: 10.1111/anae.16648 (PMC12351224; doi:10.1111/anae.16648)
Supplement: Supplementary file 1 — Appendix S1. Perioperative Quality Initiative Surgery School Consensus Group. [file ANAE-80-1115-s003.docx]

**Appendix S1: Perioperative Quality Initiative Surgery School Consensus Group**

Anna M Anderson - Research Fellow in Qualitative Research, Leeds Institute of Health Sciences, University of Leeds, Leeds, UK; Project Manager for the NIHR Leeds HealthTech Research Centre, Leeds Institute of Rheumatic and Musculoskeletal Medicine, University of Leeds, Leeds, UK and NIHR HealthTech Research Centre in Accelerated Surgical Care, Leeds, UK.

Leah Avery - Professor of Applied Health Psychology, School of Health and Life Sciences, Teesside University, Middlesbrough, Tees Valley, England. @healthpsychleah

Gabriele Baldini - Associate Professor of Anaesthesiology and Intensive Care, Department of Anesthesia and Critical Care, Azienda Ospedaliero-Universitaria Careggi, Florence, Italy. @GBaldini18

Angie Balfour - Team Lead ERAS, Stoma and Prehabilitation Services, Western General Hospital, NHS Lothian, Edinburgh, UK. @balfoor

Rachael Barlow - Clinical Lead and Consultant Allied Health Professional in Prehabilitation and Recovery, Surgical Clinical Board, Cardiff and Vale University Health Board, Cardiff, Wales. @barlowr1

Esther Carr - Senior Physiotherapist, Perioperative Medicine, South Tees NHS Foundation Trust, Middlesbrough, England

Daniel Conway - Professor of Perioperative Medicine, Manchester University NHS Foundation Trust, Manchester, UK. @periopman

Robert J Copeland - Professor of Physical Activity and Health, Advanced Wellbeing Research Centre, Sheffield Hallam University, Sheffield, England. @drrobcopeland

Anh Dang - Professor of Anaesthesia, Division of Anesthesiology, Critical Care and Pain Medicine, University of Texas MD Anderson Cancer Center, Houston, USA. @DangAqora

Andrew Davies - Consultant Upper GI Surgeon, Guy’s & St Thomas’ NHS Foundation Trust, London, UK. @adaviessurgeon

Mark R Edwards - Professor of Anaesthesia and Perioperative Medicine, NIHR Southampton Biomedical Research Centre, University Hospital Southampton NHS Foundation Trust / University of Southampton, Southampton, UK

Rochelle Furtado - Post-Doctoral Fellow, University Health Network-KITE, Toronto, Canada. @ellefurtado13

Chelsia Gillis - Assistant Professor, School of Human Nutrition, McGill University Montreal, Canada. @chelsiagillis

Hilmy Ismail - Associate Professor of Anaesthesia, Peter MacCallum Cancer Centre, Melbourne, Australia

Sandy Jack - Professor of Prehabilitation Medicine, NIHR Southampton Biomedical Research Centre, Clinical and Experimental Sciences, Faculty of Medicine, Southampton, UK. @profsandyjack

Carol Keen - Consultant Physiotherapist, Therapeutics and Palliative Care, Sheffield Teaching Hospitals NHS Foundation Trust, Sheffield, England. @Carolkeenphysio

Ruth McDonald - Senior Nurse, Elective Surgery, Women’s Health, University College London Hospitals, London, UK. @ RuthieAMcD

Scarlett McNally - Consultant Orthopaedic Surgeon, Trauma and Orthopaedics, East Sussex Healthcare NHS Trust, Eastbourne, UK. @scarlettmcnally

Zoe Merchant - Prehab4Cancer AHP Clinical Lead/GM Targeted Lung Health Check Programme Manager, North West Lung Centre, Manchester University Hospital NHS Foundation Trust, Manchester, England. @zoemerchantOT

Claire Moore - Group Clinical Director of Anaesthesia, Department of Anaesthesia, Manchester University NHS Foundation Trust Manchester Foundation Hospital NHS Trust, Manchester, England. @Clairelizmoore

John Moore - Associate Medical Director, Department of Anaesthesia, Manchester University NHS Foundation Trust, University of Manchester, Manchester, UK. @mysurgeryandme

Judith Partridge - Consultant Geriatrician, Perioperative medicine for Older People undergoing Surgery (POPS), Guy’s & St Thomas’ NHS Foundation Trust, London, UK.

Jashvant Poeran - Director of Research, Clinical Research Scientist, Department of Anesthesiology, Critical Care & Pain Management, Hospital for Special Surgery, New York, NY, USA. @jashvant_p

Brocha Z Stern - Assistant Professor, Departments of Orthopaedic Surgery and Population Health Science & Policy, Icahn School of Medicine at Mount Sinai, New York, NY, USA

Janius Tsang - Assistant Professor, Deputy Director of Prehabilitation Clinic, Department of Anesthesiology, Montreal General Hospital, McGill University Health Center, Montreal, Canada.
