# Supplementary material for: Perioperative Quality Initiative consensus statement recommendations on the definition, development, implementation and outcomes of pre‐operative surgery schools
Source: Anaesthesia. 2025 Jun 21;80(9):1115–33. doi: 10.1111/anae.16648 (PMC12351224; doi:10.1111/anae.16648)
Supplement: Supplementary file 2 — Appendix S2. Perioperative Quality Initiative grades of evidence and strength of recommendation. Appendix S3. Perioperative Quality Initiative conference structure. Appendix S4. Demographics and characteristics of expert group. Appendix S5. Behaviour change techniques used most commonly in surgery schools. Appendix S6. Suggested educational content to comply with recommendations. [file ANAE-80-1115-s002.docx]

**Appendix S2:** POQI Grades of evidence and strength of recommendation.

| **Grade of evidence** | **Definition** |
| --- | --- |
| A | **High Quality**: Further research is very unlikely to change our confidence in the estimate of the effect |
| B | **Moderate Quality**: Further research is likely to have an important impact on our confidence in the estimate of effect and may change the estimate |
| C | **Low Quality**: Further research is very likely to have an important impact on our confidence in the estimate of effect and is likely to change the estimate |
| D | **Very Low Quality**: Any estimate of effect is very uncertain |
| **Strength of Recommendation** | **Definition** |
| Strong | **For patients**—most people in your situation would want the recommended course of action and only a small proportion would not; request discussion if the intervention is not offered.  **For clinicians**—most patients should receive the recommended course of action.  **For policymakers**—the recommendation can be adopted as a policy in most situations. |
| Weak | **For patients**—most people in your situation would want the recommended course of action, but many would not.  **For clinicians**—you should recognize that different choices will be appropriate for different patients and that you must help each patient to arrive at a management decision consistent with her or his values and preferences.  **For policymakers**—policy making will require substantial debate and involvement of many stakeholders. |

Adapted from Guyatt et al. [17]

**Appendix S3:** POQI Conference structure

| **Date/Duration** | **Title** | **Content** |
| --- | --- | --- |
| 5 January 2024  2 h | Preliminary meet | Introductions for all experts  Presentation on background to surgery schools  Introduction of proposed draft statements and recommendations. Explanation of GRADE and POQI rules. |
| 10 January 2024  3 h | Workshop 1 | **0.5 h plenary session** All experts reviewing all statements and recommendations.  **1.5 h split into group 1 and 2** Discussion and voting on statements and recommendations following issues raised in plenary session.  **1 h plenary session** Feedback on modifications made to statements and recommendations and justification given. |
| 29 January 2024  4 h | Workshop 2 | **1.5 h plenary session.** All experts to review all statements and recommendations and identify any disagreement for workshop discussion.  **1.5 h split into group 1 and 2.** Discussion and voting on statements and recommendations following issues raised in plenary session.  **1 h plenary session.** Feedback on modifications made to statements and recommendations and justification given. |
| 19 February 2024  1.5 h | Patient and public involvement meeting | Presentation of all draft statements and recommendations followed by open discussion. |
| 26 February  3 h | Finalise and voting | **Plenary session** Feedback from PPI group. Finalise wording, and GRADE.  Final voting on wording, grading and strength of recommendation of statements and recommendations. |

**Appendix S4:** Demographics and characteristics of expert group

| Male / Female / Other | n=12 / n=20 / n=0 |
| --- | --- |
| **Workplace location:**  England  Wales  Scotland  Italy  USA  Canada  Australia | n=20  n=1  n=1  n=1  n=5  n=3  n=1 |
| **Profession**  Nurse  Physiotherapist  Occupational Therapist  Dietitian  Anaesthetist  Psychologist  Clinician Scientist  Geriatrician  Epidemiologist | n=3  n=4  n=2  n=2  n=13  n=2  n=3  n=1  n=1  n=1 |
| **Years of experience as health/academic professional:**  0-9  10-19  20-29  30-39 | n=1  n=11  n=12  n=8 |
| **Primary workplace setting:**  State funded hospital  Private hospital  Educational establishment | n=21  n=3  n=8 |
| **Currently providing preoperative education to patients as part of clinical role.** | Yes - n=23  No- n= 3  N/A n = 6 |
| **How would you describe your ethnicity?**  White British  White other  Asian  South Asian  Asian other | n=21  n=4  n=4  n=2  n=1 |

**Appendix S5:**  Most commonly used behaviour change techniques in surgery schools

| **Behaviour Change Technique** |
| --- |
| Information about health & emotional consequences [29,36,78] |
| Instruction / demonstration on how to perform a behaviour [29,36,78] |
| Behavioural practice [36,78] |
| Goal setting, action planning, self-monitoring [29,36,78,127] |
| Review behaviour goals [36,78] |
| Behaviour substitution [29] |
| Delivered by a credible source [29,36] |
| Strategies to promote habit formation [36,78] |
| Social support [29,36,78] |
| Verbal persuasion about capability [36] |
| Discrepancy between current behaviour and goal [36] |
| Setting graded tasks [36,78] |

*Please see main document for references

**Appendix S6 :** Suggested educational content to comply with recommendations

| **Recommendation** | **Suggested Content** |
| --- | --- |
| Surgery schools should help set expectations and develop a partnership with patients in preparation for and recovery from surgery. | Describe the journey from preoperative care through to admission and discharge. This may include:   - Preoperative tests - Preparing to come into hospital - What to bring to hospital - Oral carbohydrate loading and food and water intake prior to surgery - Day of surgery - Possible complications - Critical care/enhanced care units/ ward routines - Pain management - Enhanced recovery - Discharge home - Self-care and rehabilitation |
| Surgery school should support patients to understand the importance of optimising long-term health conditions prior to surgery and signpost to appropriate resources. | - Describe the impact of chronic health conditions on recovery from surgery. - Suggestions for optimisation: Investigating new-onset undiagnosed symptoms, recent review of known chronic conditions. - Signposting for particular conditions including cardiac and respiratory disease, diabetes and frailty. |
| Surgery schools should support patients to understand common postoperative complications. | - Describe most common complications including pulmonary complications, infections, thrombosis, kidney injury and delirium. |
| Surgery schools should inform patients about the risks of pulmonary complications and give advice on activities to reduce this risk. | - Explain why pulmonary complications may occur following surgery and general anaesthesia. - Describe the impact of pulmonary complications on recovery. - Outline what patients can do to reduce their risks e.g. stop smoking and vaping, regular postoperative mobility, thorough mouth care, deep breathing and coughing. - Demonstrate and practice active cycle of breathing techniques. |
| Surgery schools should support patients to understand the principles of multi-modal prehabilitation and the impact of health behaviours on surgical outcomes. | - Describe multimodal prehabilitation and how it works. - Describe the impact of prehabilitation on reducing the risk complications and improved the health outcomes that can result from better health behaviours. |
| Surgery schools should support patients to plan and undertake physical activity and exercise. | - Increase physical activity in line with WHO guidelines [108]. - Reduce sedentary time. - Advise patients to increase levels of aerobic exercise and provide examples e.g. brisk walking / cycling/ running/ dancing. - Explain how to identify moderate/high intensity exercise (Borg scale (10 point); sing test). - Explain principles of interval training: e.g. walk fast for 2 minutes, then walk slowly for 3 minutes. - Encourage patients to choose their preferred exercise, set their own S.M.A.R.T. goals and identify barriers to be overcome. - Advise on when to resume exercise after surgery and rehabilitation advice. - Refer / signpost to available supportive resources/exercise referral schemes/prehabilitation services. - Provide examples of strength training. - Recommend weekly strength / resistance training. |
| Surgery schools should emphasise the importance of good nutrition before and after surgery and signpost to appropriate resources. | - Provide information on the importance of eating well before, during and after surgery. - Describe what eating well looks like – providing visual examples, all food groups, balanced meals, adequate protein, avoid ultra processed foods, promote adequate fiber intake by consuming whole grains, fruits, and vegetables. - Inform patients on how they can identify if they are at risk of malnutrition (Loss of appetite, eating less than usual, unintentional weight loss) and what they should do about this. - Provide generic advice on gaining and maintaining weight (for those at risk of malnutrition). - Suggest actions to take (e.g., connect with community resources) if BMI over > 40 to support controlled weight loss before and after surgery. - Provide tips for eating well in hospital and post-discharge signpost/referral to community or primary care services for further management of malnutrition (as needed), or for long term management of obesity. - Signposting to specialty specific health eating resources. |
| Surgery schools should support patients to prepare psychologically for surgery | - Prepare patients to expect a mental and physical challenge - Validate and normalise the anxiety that often comes with that having surgery. - Help patients to identify if they have any of the key symptoms and signs of anxiety and/or depression and when to seek professional help. - Suggest regular wellbeing activities such as relaxation, exercise and avoidance of alcohol. - Sign-post to additional wellbeing resources. |
| Surgery schools should inform patients about the risks of excessive alcohol consumption and support patients to limit alcohol intake prior to surgery. | - Describe the potential impact of excessive alcohol intake on postoperative recovery. - Provide an opportunity for patients to self-screen their alcohol drinking habits. - Recommend cessation 2-4 weeks prior to surgery. - Advise those drinking alcohol daily of the risk of withdrawal and suggest strategies for avoiding it. - Elicit suggestions on how alcohol intake could be reduced. Thereby encouraging patients to develop their own strategy for reducing alcohol intake preoperatively. |
| Surgery schools should inform patients about the benefits of stopping smoking before surgery and support patients to stop smoking. | - Describe the potential impact of smoking on surgical recovery. - Advise patients to stop smoking as soon as possible and highlight the overall benefits of doing so. - Sign-post to smoking cessation services. |
| Surgery schools should support patients to understand how their pain will be managed around the time of surgery. | - Describe what to expect with regard to pain and discomfort following surgery. - Describe types of pain (including preoperative chronic pain) and the common analgesia modalities used to manage postoperative pain (e.g. Patient Controlled Analgesia, epidural etc.) - Describe the impact of pain on lung function. - Inform patients on when to ask for more pain relief. - Describe how the side effects of analgesia can be managed. - Describe non-pharmacological approaches to pain relief. |
| Surgery schools should inform patients about the principles of Enhanced Recovery and their role in early drinking, eating and mobilization (DrEaMing) | - Describe how the principles of enhanced recovery improve recovery. - Explain the importance of DrEaMing. - Describe the patient’s role in recovery. |
