# Supplementary material for: Perioperative Quality Initiative consensus statement recommendations on the definition, development, implementation and outcomes of pre‐operative surgery schools
Source: Anaesthesia. 2025 Jun 21;80(9):1115–33. doi: 10.1111/anae.16648 (PMC12351224; doi:10.1111/anae.16648)
Supplement: Supplementary file 3 — Plain Language Summary [file ANAE-80-1115-s001.docx]

**Plain Language Summary**

Before big, planned operations, many hospitals now run special group classes called ‘surgery schools.’ These help patients learn what to expect and how to get ready for their operation. But doctors still don’t know for sure how well these classes work, and there is no clear guide as to how they should be set up. To help fix this, a group of experts from around the world worked together. They wanted to agree on what surgery schools should look like and what they should teach. They met online in meetings and looked at lots of studies and research. They talked through ideas in several rounds until they all agreed on the best advice. The team agreed on a definition of what a surgery school is and produced 21 pieces of advice about how to plan and run these schools. These included what should be taught, how to make the classes easy for everyone to join and how to deliver them well. They also found 17 topics that need more research. These new ideas can help doctors and hospital staff create better surgery schools. This might make the classes more useful and more similar across different hospitals. The team also hopes their work will help guide health leaders when they make rules and decisions about these programmes.
